# High-intensity interval training versus moderate-intensity continuous training for polycystic ovary syndrome: a meta-analysis of randomized controlled trials

**DOI:** 10.3389/fendo.2025.1672257

**Published:** 2025-10-16

**Authors:** Yi Zhao, Yu Long, Hongjiao Zhu, Run He, Yunxia Chen, Jingrong Li

**Affiliations:** ^1^ Reproductive Medicine Center, Dazhou Central Hospital, Dazhou, China; ^2^ Department of Gynecology, Dazhou Central Hospital, Dazhou, China

**Keywords:** polycystic ovary syndrome, high-intensity interval training, moderate-intensity continuous training, meta-analysis, randomized controlled trials

## Abstract

**Objective:**

Polycystic Ovary Syndrome (PCOS) is the most prevalent endocrine disorder affecting women of reproductive age. Lifestyle modifications, particularly exercise, are cornerstone management strategies, with High-Intensity Interval Training (HIIT) and Moderate-Intensity Continuous Training (MICT) being commonly recommended modalities. Despite their widespread use, high-quality evidence directly comparing HIIT and MICT in women with PCOS is limited. This meta-analysis aims to rigorously compare the effects of HIIT versus MICT in women with PCOS to provide precise and robust evidence for clinical recommendations.

**Methods:**

This meta-analysis adhered to PRISMA guidelines, conducting a comprehensive search across PubMed, EMBASE, Web of Science, and Cochrane Library databases up to April 15, 2025. Randomized controlled trials (RCTs) directly comparing supervised HIIT and MICT interventions of at least 12 weeks in premenopausal women (18–50 years) diagnosed with PCOS were included. Outcome data covered anthropometric measures, cardiorespiratory fitness, glucose and insulin metabolism, lipid profile, and hormonal parameters. Methodological quality was assessed using the Cochrane Risk of Bias Tool (RoB 2), and overall evidence certainty was determined via GRADE methodology. Statistical analyses were performed using Review Manager (RevMan) 5.4.1, with continuous variables analyzed as Weighted Mean Differences (WMD) with 95% Confidence Intervals (CIs).

**Results:**

A total of six RCTs were included in the meta-analysis. The main findings indicate no statistically significant superiority of either HIIT or MICT across anthropometric outcomes (weight, BMI, waist circumference, hip circumference, WHR), cardiorespiratory fitness (VO2max, SBP, DBP), glucose and insulin metabolism (fasting glucose, fasting insulin, HOMA-IR), lipid profile (total cholesterol, HDL cholesterol, LDL cholesterol, triglycerides), or hormonal parameters (testosterone, SHBG, FAI). The certainty of evidence for these outcomes ranged from very low to low.

**Conclusion:**

Based on the current low to very low certainty evidence from RCTs, there is no statistically significant superiority of HIIT over MICT for improving anthropometric, cardiorespiratory, metabolic, or hormonal outcomes in women with PCOS. These findings suggest that either HIIT or MICT can be recommended based on patient preference, but larger RCTs are needed due to low evidence certainty. This study received no funding.

## 1 Introduction

Polycystic Ovary Syndrome (PCOS) is a common and complex endocrine disorder, characterized by hyperandrogenism, ovulatory dysfunction, and polycystic ovaries (1, 2). With an estimated prevalence of 11-13% among women worldwide (3), PCOS represents a significant public health and economic burden (4, 5). PCOS stands as the most prevalent endocrine disorder affecting women of reproductive age (6). Its diverse clinical presentation encompasses reproductive dysfunctions like infertility and menstrual irregularities, alongside significant metabolic complications including insulin resistance, type 2 diabetes, cardiovascular disease, and obesity (1, 2). Critically, PCOS is the leading cause of anovulatory infertility (7), and a substantial contributor to early-onset type 2 diabetes and various psychological disorders (8). The chronic nature of PCOS and its associated comorbidities necessitate effective and sustainable management strategies.

The underlying pathophysiology of PCOS is multifactorial, involving a complex interplay of genetic predispositions and environmental factors. Key mechanisms include insulin resistance, compensatory hyperinsulinemia, hyperandrogenism, chronic low-grade inflammation, and altered adipose tissue function (1, 2). Current non-pharmacological management recommendations for PCOS primarily focus on lifestyle modifications, including dietary interventions and regular exercise, to address these core pathological features (3). Exercise is recognized as a cornerstone of PCOS management due to its ability to improve insulin sensitivity, reduce androgen levels, mitigate inflammation, enhance body composition, and improve cardiovascular health (9–11).

Among the various exercise modalities, High-Intensity Interval Training (HIIT) and Moderate-Intensity Continuous Training (MICT) are two commonly recommended approaches (3). Typically, HIIT involves bursts at 80-95% of maximum heart rate (HRmax) for 30 seconds to 4 minutes, alternated with recovery at 50-60% HRmax, for 20–40 minutes per session, 3–5 times weekly. MICT entails continuous exercise at 50-70% HRmax for 30–60 minutes per session at similar frequencies (9, 11). HIIT offers notable time efficiency, potentially achieving comparable physiological benefits in less total exercise time, and its perceived enjoyability relative to MICT further contributes to its enhanced adherence potential. Both exercise patterns have been shown to improve insulin sensitivity, reduce hyperandrogenism, exert anti-inflammatory effects, improve body composition, and enhance cardiorespiratory fitness in women with PCOS (12, 13).

Despite the growing interest in exercise as a therapeutic intervention for PCOS, there remains limited high-quality evidence directly comparing the effects of HIIT and MICT head-to-head. Previous systematic reviews, often limited by small sample sizes and the inclusion of broad exercise interventions or non-direct comparisons, have yielded diverse and sometimes inconclusive findings regarding the relative superiority of one modality over another for specific PCOS outcomes (14–17). Therefore, a comprehensive and updated meta-analysis exclusively focusing on randomized controlled trials (RCTs) directly comparing HIIT and MICT in women with PCOS is critically needed to provide precise and robust evidence for clinical recommendations.

This meta-analysis aims to rigorously compare the effects of HIIT versus MICT in women with PCOS. We hypothesize that HIIT and MICT will lead to comparable improvements in PCOS-related outcomes.

## 2 Methods

### 2.1 Study design and registration

This meta-analysis adhered to the Preferred Reporting Items for Systematic Reviews and Meta-Analyses (PRISMA) guidelines (18) and was preregistered in the International Prospective Register of Systematic Reviews (PROSPERO) with the registration ID: CRD42025649165. This study received no funding, and all authors declared no conflict of interest. Two independent reviewers (YZ and YL) conducted the literature search, extracted data, assessed the methodological quality of included studies, and performed statistical analyses, with any discrepancies resolved through discussion with a third reviewer (JL).

### 2.2 Literature search

A comprehensive search was conducted across PubMed, EMBASE, Web of Science, and Cochrane Library databases, including all records available up to April 15, 2025. The search strategy used the following main search terms: (“high-intensity interval training” OR “HIIT” OR “high-intensity intermittent exercise” OR “interval training”) AND (“moderate-intensity continuous training” OR “MICT” OR “moderate-intensity exercise” OR “continuous aerobic training”) AND (“polycystic ovary syndrome” OR “PCOS” OR “polycystic ovarian syndrome” OR “hyperandrogenic anovulation”). Terms like ‘intermittent’ and ‘interval’ were included to account for synonymous usage in literature, while ‘continuous aerobic’ captured MICT variants. Screening ensured conceptual consistency by verifying that HIIT protocols featured high-intensity bursts (typically >80% HRmax or equivalent) with recovery, and MICT involved moderate continuous exercise (typically 50-75% HRmax), though we accommodated minor variations as per study reporting to reflect practical implementations. Criteria were applied consistently, with all studies verified for direct HIIT/MICT comparisons. Search queries were tailored to meet the specific requirements of each database (**Supplementary Table 1**).

### 2.3 Inclusion and exclusion criteria

Inclusion criteria: (1) studies involving premenopausal women aged 18 to 50 years, diagnosed with PCOS per the Rotterdam Criteria (19); (2) RCTs directly comparing HIIT with MICT as supervised exercise interventions lasting at least 12 weeks, ensuring consistency in all other variables such as diet and medication except the exercise protocol; (3) studies reporting at least one outcome with baseline and endpoint data, covering anthropometric measures, cardiorespiratory fitness, glucose and insulin metabolism, lipid profile, or hormonal parameters.

Exclusion criteria: (1) studies including pregnant participants; (2) studies involving participants using antihypertensive medications, insulin sensitizers, dietary supplements, weight loss medications, or hormonal contraceptives within 3 months prior to enrolment; (3) non-English studies; (4) studies incorporating additional forms of exercise beyond HIIT or MICT.

### 2.4 Data extraction

Extracted study details included the first author’s name, publication year, study location, sample size, patient demographics (age and body mass index [BMI]), PCOS condition, and specifics of both HIIT and MICT intervention. Outcome data were derived as the difference (Δ) between endpoint and baseline values (endpoint values – baseline values) for comparisons between groups, categorized into five major domains: anthropometric measures, cardiorespiratory fitness, glucose and insulin metabolism, lipid profile, and hormonal parameters.

Anthropometric measures included body mass index (BMI in kg/m²), waist and hip circumference (in cm), as well as waist-to-hip ratio (WHR). Cardiorespiratory fitness included maximal oxygen uptake (VO_2_max in ml/kg/min), systolic blood pressure (SBP) and diastolic blood pressure (DBP) in mm Hg. Glucose and insulin metabolism included fasting glucose (in mmol/L), fasting insulin (in µIU/mL), and homeostatic model assessment of insulin resistance (HOMA-IR). Lipid profile comprised total cholesterol (in mmol/L), high-density lipoprotein cholesterol (HDL cholesterol in mmol/L), low-density lipoprotein cholesterol (LDL cholesterol in mmol/L), and triglycerides (in mmol/L). Hormonal parameters included testosterone (in nmol/L), sex hormone-binding globulin (SHBG in nmol/L), and free androgen index (FAI).

### 2.5 Quality assessment

The methodological quality of included studies was appraised using the revised Cochrane Risk of Bias Tool (RoB 2) for RCTs (20). This tool evaluated bias across five domains: randomization process, deviations from intended interventions, missing outcome data, measurement of the outcome, and selection of the reported result. Each domain was classified as low risk, high risk, or some concerns based on RoB 2 guidelines. The overall evidence certainty was determined using the Grading of Recommendations, Assessment, Development and Evaluation (GRADE) methodology, factoring in risk of bias, inconsistency, indirectness, imprecision, and publication bias, with quality rated as high, moderate, low, or very low. Publication bias was assessed via funnel plots and Egger’s test for outcomes with ten or more studies.

### 2.6 Statistical analysis

Meta-analyses were performed using Review Manager (RevMan) version 5.4.1 (The Cochrane Collaboration, Oxford, UK). Continuous variables were analyzed as Weighted Mean Differences (WMD) with 95% Confidence Intervals (CIs), while dichotomous variables were expressed as pooled Odds Ratios (ORs) with 95% CIs. Heterogeneity was evaluated using Cochrane’s Q test and the I² statistic. For analyses with I² <50%, a fixed-effects model was employed, whereas a random-effects model was adopted for I² ≥50%. Forest plots were used to present the pooled effect sizes, with statistical significance defined as P <0.05.

## 3 Results

### 3.1 Study selection and characteristics

**Figure 1:**
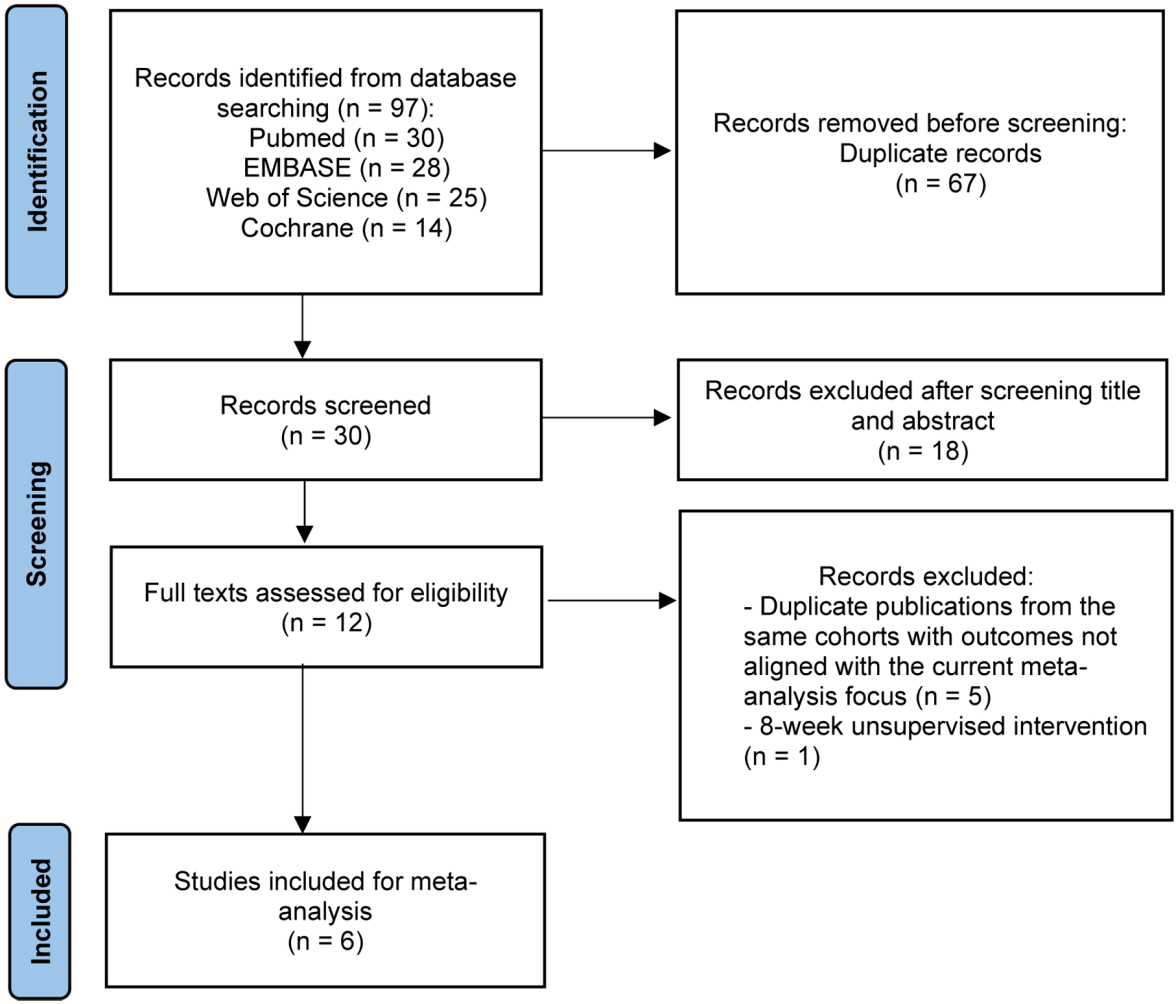
PRISMA flow chart of literature retrieval.

A search of PubMed, EMBASE, The Cochrane Library, and Web of Science found 97 studies. After removing 67 duplicates, 30 studies were screened by title and abstract. Of these, 18 were excluded, leaving 12 studies for full-text review and reference checking. After reviewing the full texts, 6 studies were excluded, with 5 (21–25) excluded due to being duplicate publications or secondary analyses of the same cohort (retaining only the initial study with the most comprehensive outcome data) and 1 (26) excluded due to an 8-week unsupervised home-based HIIT and MICT intervention. Finally, a total of 6 RCTs (27–32) were included (**Figure 1**). The main details of these studies are presented in **Table 1** and **Table 2**.

**Table 1:** Characteristics of the included studies.

First author	Year	Study location	Patients, n		Age, y Mean ± SD or Median (IQR)		BMI, kg/m^2^ Mean ± SD or Median (IQR)		PCOS condition
			HIIT	MICT	HIIT	MICT	HIIT	MICT	
Lopes	2018	Brazil	22	23	29.4 ± 4.1	30.2 ± 5.1	29.0 ± 4.8	29.3 ± 5.6	No diabetes, Rotterdam phenotypes 1–4, stratified by BMI (< 30 and ≥30)
Ribeiro	2020	Brazil	29	28	29.0 ± 4.3	29.1 ± 5.3	28.7 ± 4.8	28.4 ± 5.6	No diabetes, Rotterdam phenotypes 1–4, stratified by BMI (< 30 and ≥30)
Benham	2021	Canada	16	14	29.1 ± 4.1	29.5 ± 4.6	31.4 ± 8.6	31.3 ± 9.0	No diabetes, Rotterdam phenotypes 1–4, stratified by BMI (< 28 and ≥28)
Aktas	2022	Turkey	10	10	25.1 ± 4.6	24.6 ± 6.7	28.7 ± 6.9	28.6 ± 4.9	No diabetes, Rotterdam criteria, severity not quantified
Patten	2022	Australia	15	14	29.7 ± 4.8	32.5 ± 6.2	35.5 ± 6.8	35.6 ± 7.0	No diabetes, Rotterdam phenotypes 1–4, BMI >25
Philbois	2022	Brazil	25	25	29 ± 4	29 ± 5	27.8 ± 4.2	27.7 ± 5.7	No diabetes, Rotterdam phenotypes 1–4

HIIT, high-intensity interval training; MICT, moderate-intensity continuous training; n, number; SD, standard deviation; IQR, interquartile range; BMI, body mass index; PCOS, polycystic ovary syndrome.

**Table 2:** Characteristics of training protocols.

First author	Year	Group	Duration (weeks)	Frequency (times/week)	Intensity	Duration/session (min)	Supervision	Equipment
Lopes	2018	HIIT	16	3	65-80% HRmax	30	Supervised by training team	Treadmills (Embreex 570-L/Pro), Polar RS 810
		MICT	16	3	65 HRmax	30	Supervised by training team	Treadmills (Embreex 570-L/Pro), Polar RS 810
Riberiro	2020	HIIT	16	3	85–90% HRmax (2 min)/65–70% HRmax (3 min)	35–45	Supervised by sport scientists	Treadmills (Embreex 570-L/Pro), Polar RS800CX
		MICT	16	3	70–80% HRmax	50	Supervised by sport scientists	Treadmills (Embreex 570-L/Pro), Polar RS800CX
Benham	2021	HIIT	24	3	90% HRR (30 sec)/low (90 sec), 10 cycles	20–30	Partially supervised (2 times/week) + self-monitored	Participant-chosen aerobic equipment, Polar A370, Polar H10
		MICT	24	3	50–60% HRR	40	Partially supervised (2 times/week) + self-monitored	Participant-chosen aerobic equipment, Polar A370, Polar H10
Aktas	2022	HIIT	12	3	2 min running alternated with walking	30	Implied supervision by research team	Not specified
		MICT	12	3	Moderate tempo running	30	Implied supervision by research team	Not specified
Patten	2022	HIIT	12	3	90–100% HRpeak (1 min) or 90–95% HRpeak (4 min) with active recovery	30-40	Supervised by accredited exercise physiologists	Stationary cycle ergometer, Polar H10
		MICT	12	3	60–75% HRpeak	45	Supervised by accredited exercise physiologists	Stationary cycle ergometer, Polar H10
Philbois	2022	HIIT	16	3	85–90% HRR (2 min)/65–70% HRR (3 min)	35–45	Supervised by professional team	Motorized treadmill, Polar RS810
		MICT	16	3	70–80% HRR	50	Supervised by professional team	Motorized treadmill, Polar RS810

HIIT, high-intensity interval training; MICT, moderate-intensity continuous training; HRmax, maximum heart rate; HRR, heart rate reserve; HRpeak, Peak Heart Rate; m, minutes; w, weeks.

### 3.2 Quality assessment

**Figure 2:**
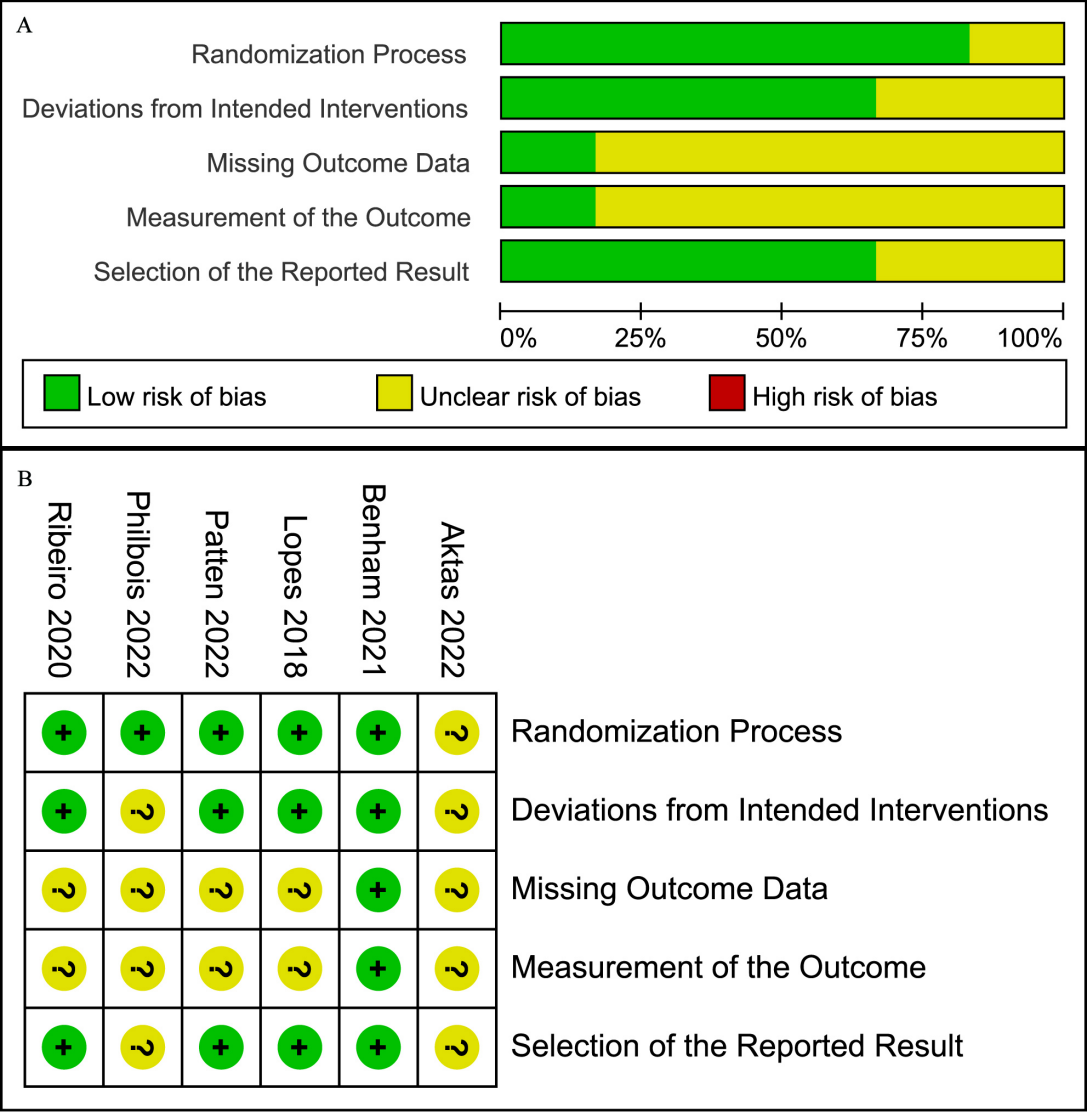
Risk of bias graph **(A)** Graph of the risk of bias summary for the included studies, **(B)** Graph of the risk of bias for each included study.

The quality of the included studies was assessed using the RoB 2 criteria, as illustrated in **Figure 2**, with no study exhibiting high risk in any domain. The primary “some concerns” were concentrated in the areas of missing outcome data and measurement of the outcomes. Overall, the quality of the included studies was considered moderate.

### 3.3 Anthropometric measures

**Figure 3:**
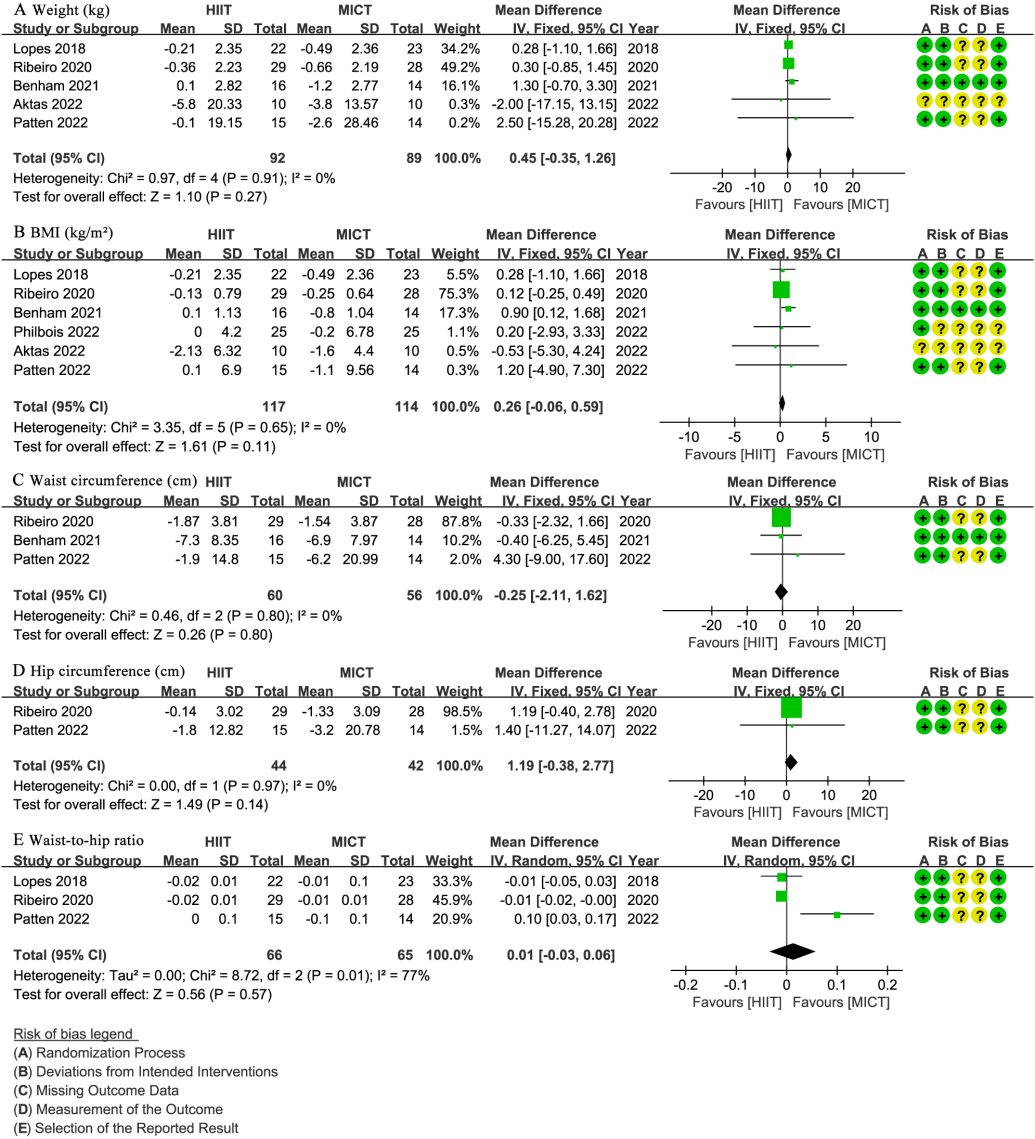
Meta-analysis of: **(A)** weight (kg), **(B)** body mass index (BMI) (kg/m2), **(C)** waist circumference (cm), **(D)** hip circumference (cm), **(E)** waist-to-hip ratio (WHR).

Five studies (27–30, 32) involving 181 patients reported body weight (kg). The results showed no significant difference in weight change between the HIIT and MICT groups (WMD: 0.45; 95% CI: -0.35 to 1.26; I² = 0%; P = 0.27) (**Figure 3A**). Similarly, six studies (27–32) with 231 patients reported BMI (kg/m^2^), indicating no significant difference between groups (WMD: 0.26; 95% CI: -0.06 to 0.59; I² = 0%; P = 0.11) (**Figure 3B**). Three studies (28, 30, 32) involving 116 patients assessed waist circumference (cm), showing no significant difference between the HIIT and MICT groups (WMD: -0.25; 95% CI: -2.11 to 1.62; I² = 0%; P = 0.80) (**Figure 3C**). Two studies (30, 32) with 86 patients reported hip circumference (cm), with no significant difference observed (WMD: 1.19; 95% CI: -0.38 to 2.77; I² = 0%; P = 0.14) (**Figure 3D**). Three studies (29, 30, 32) involving 131 patients reported WHR, showing no significant difference (WMD: 0.01 higher; 95% CI: -0.03 to 0.06; I² = 77%; P = 0.57) (**Figure 3E**).

### 3.4 Cardiorespiratory fitness

**Figure 4:**
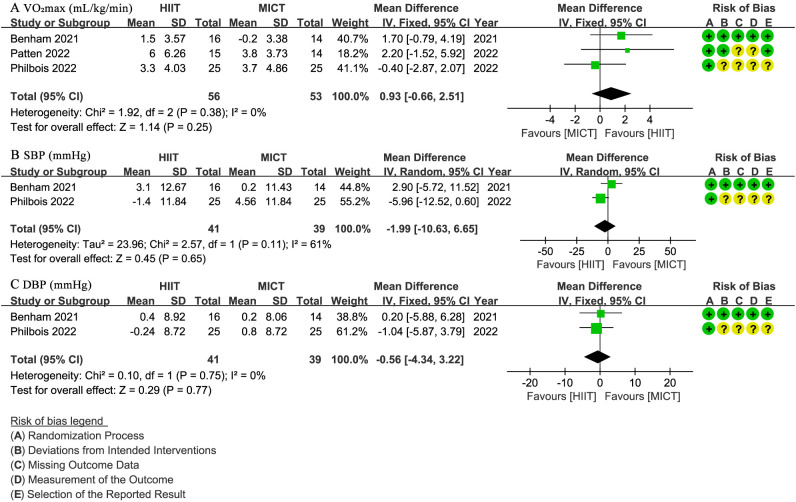
Meta-analysis of: **(A)** VO2max (mL/min/kg), **(B)** systolic blood pressure (SBP) (mmHg), **(C)** diastolic blood pressure (DBP) (mmHg).

Three studies (28, 30, 31) with 109 patients evaluated VO_2_max (mL/min/kg), indicating no significant difference between groups (WMD: 0.93; 95% CI: -0.66 to 2.51; I² = 0%; P = 0.25) (**Figure 4A**). Two studies (28, 31) involving 80 patients reported SBP and DBP (mmHg), with no significant difference observed for SBP (WMD: -1.99; 95% CI: -10.63 to 6.65; I² = 61%; P = 0.65) (**Figure 4B**) or DBP (WMD: -0.56; 95% CI: -4.34 to 3.22; I² = 0%; P = 0.77) (**Figure 4C**).

### 3.5 Glucose and insulin metabolism

**Figure 5:**
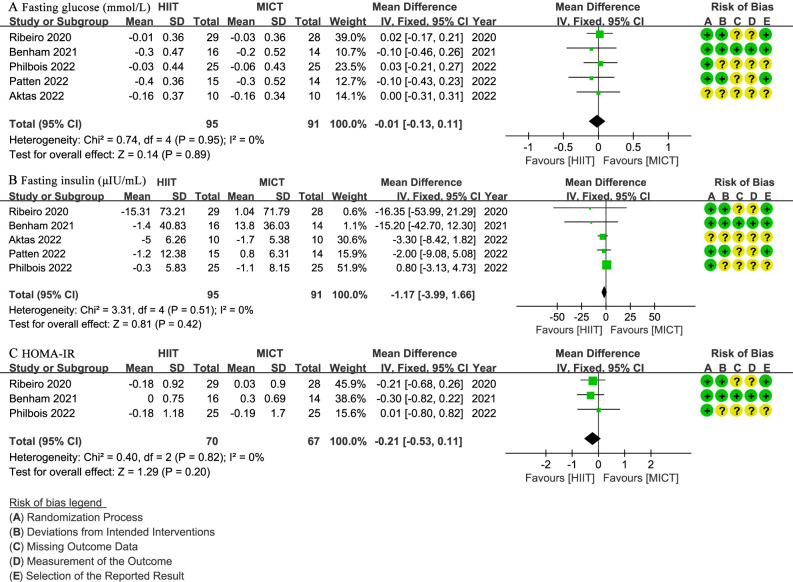
Meta-analysis of: **(A)** fasting glucose (mmol/L), **(B)** fasting insulin (µIU/mL), **(C)** homeostatic model assessment for insulin resistance (HOMA-IR).

Five studies (27, 28, 30–32) involving 186 patients assessed fasting glucose (mmol/L), indicating no significant difference between the HIIT and MICT groups (WMD: -0.01; 95% CI: -0.13 to 0.11; I² = 0%; P = 0.89) (**Figure 5A**). The same five studies (27, 28, 30–32) reported fasting insulin (µIU/mL), with no significant difference observed (WMD: -1.17; 95% CI: -3.99 to 1.66; I² = 0%; P = 0.42) (**Figure 5B**). Three studies (28, 31, 32) with 137 patients evaluated HOMA-IR, showing no significant difference (WMD: -0.21; 95% CI: -0.53 to 0.11; I² = 0%; P = 0.20) (**Figure 5C**).

### 3.6 Lipid profile

**Figure 6:**
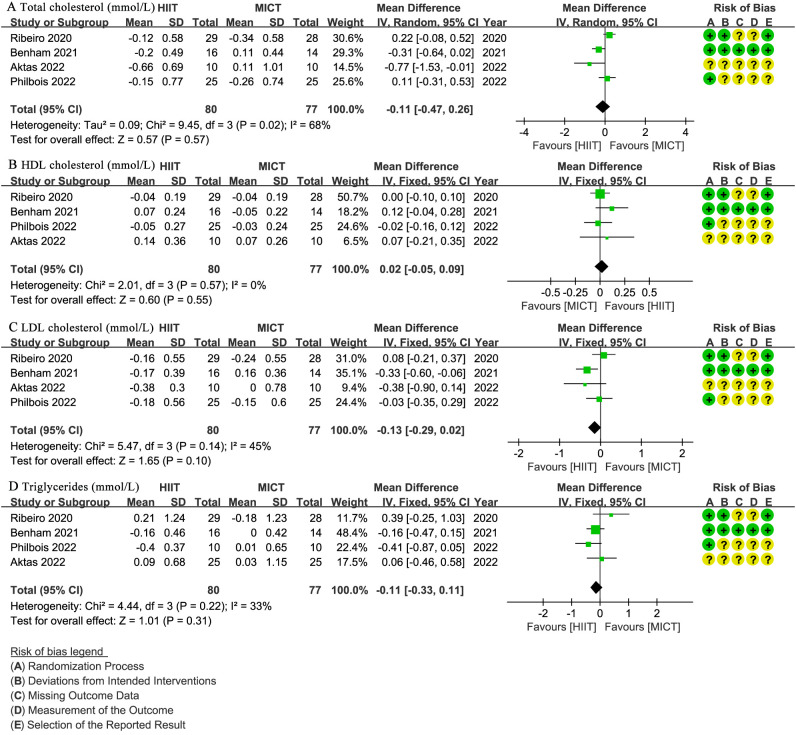
Meta-analysis of: **(A)** total cholesterol (mmol/L), **(B)** high-density lipoprotein (HDL) cholesterol (mmol/L), **(C)** low-density lipoprotein (LDL) cholesterol (mmol/L), **(D)** triglycerides (mmol/L).

Four studies (27, 28, 31, 32) involving 157 patients reported total cholesterol (mmol/L), HDL cholesterol (mmol/L), LDL cholesterol (mmol/L), and triglycerides (mmol/L) (**Figure 6**). There was no significant difference between groups for total cholesterol (WMD: -0.11; 95% CI: -0.47 to 0.26; I² = 68%; P = 0.57) (**Figure 6A**), HDL cholesterol (WMD: 0.02; 95% CI: -0.05 to 0.09; I² = 0%; P = 0.55) (**Figure 6B**), LDL cholesterol (WMD: -0.13; 95% CI: -0.29 to 0.02; I² = 45%; P = 0.10) (**Figure 6C**), or triglycerides (WMD: -0.11; 95% CI: -0.33 to 0.11; I² = 33%; P = 0.31) (**Figure 6D**).

### 3.7 Hormonal parameters

**Figure 7:**
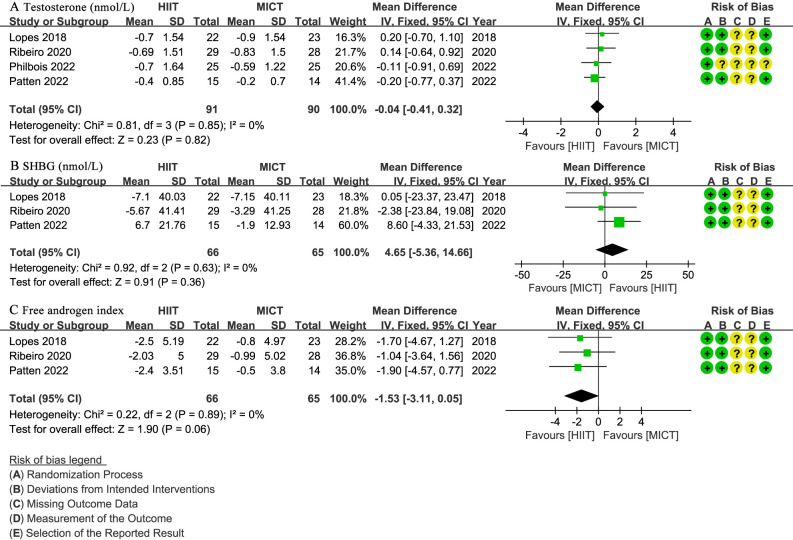
Meta-analysis of: **(A)** testosterone levels (nmol/L), **(B)** sex hormone binding globulin (SHBG) (nmol/L), **(C)** free testosterone index (FAI).

Four studies (29–32) involving 181 patients reported testosterone levels (nmol/L), with no significant difference observed (WMD: -0.04; 95% CI: -0.41 to 0.32; I² = 0%; P = 0.82) (**Figure 7A**). Three studies (29, 30, 32) with 131 patients assessed SHBG (nmol/L) and FAI, showing no significant difference for SHBG (WMD: 4.65; 95% CI: -5.36 to 14.66; I² = 0%; P = 0.36) (**Figure 7B**) or FAI (WMD: -1.53; 95% CI: -3.11 to 0.05; I² = 0%; P = 0.06) (**Figure 7C**).

### 3.8 Quality of evidence

The GRADE methodology was applied to assess the certainty of evidence for each outcome (**Table 3**). The quality of evidence was very low for WHR, SBP, total cholesterol, and low for other outcomes.

**Table 3:** GRADE summary of findings.

HIIT versus MICT for PCOS patients				
Patient or population: PCOS Intervention: HIIT training Comparison: MICT training				
Outcomes	Effect estimate	95% CI	No of Participants (studies)	Quality of the evidence (GRADE)
Weight (kg)	WMD: 0.45 higher in the interventional group.	0.35 lower to 1.26 higher	181 (5 studies)	⊕⊕⊝⊝ **low**
BMI (kg/m^2^)	WMD: 0.26 higher in the interventional group.	0.06 lower to 0.59 higher	231 (6 studies)	⊕⊕⊝⊝ **low**
Waist circumference (cm)	WMD: 0.25 lower in the interventional group.	2.11 lower to 1.62 higher	116 (3 studies)	⊕⊕⊝⊝ **low**
Hip circumference (cm)	WMD: 1.19 higher in the interventional group	0.38 lower to 2.77 higher	86 (2 studies)	⊕⊕⊝⊝ **low**
WHR	WMD: 0.01 higher in the interventional group.	0.03 lower to 0.06 higher	131 (3 studies)	⊕⊝⊝⊝ **very low**
VO_2max_ (mL/kg/min)	WMD: 0.93 higher in the interventional group.	0.66 lower to 2.51 higher	109 (3 studies)	⊕⊕⊝⊝ **low**
SBP (mmHg)	WMD: 1.99 lower in the interventional group.	10.63 lower to 6.65 higher	80 (2 studies)	⊕⊝⊝⊝ **very low**
DBP (mmHg)	WMD: 0.56 lower in the interventional group.	4.34 lower to 3.22 higher	80 (2 studies)	⊕⊕⊝⊝ **low**
Fasting glucose (mmol/L)	WMD: 0.01 lower in the interventional group.	0.13 lower to 0.11 higher	186 (5 studies)	⊕⊕⊝⊝ **low**
Fasting insulin (μIU/mL)	WMD: 1.17 lower in the interventional group.	3.99 lower to 1.66 higher	186 (5 studies)	⊕⊕⊝⊝ **low**
HOMA-IR	WMD: 0.21 lower in the interventional group.	0.53 lower to 0.11 higher	137 (3 studies)	⊕⊕⊝⊝ **low**
Total cholesterol (mmol/L)	WMD: 0.11 lower in the interventional group.	0.47 lower to 0.26 higher	157 (4 studies)	⊕⊝⊝⊝ **very low**
HDL cholesterol (mmol/L)	WMD: 0.02 higher in the interventional group.	0.05 lower to 0.09 higher	157 (4 studies)	⊕⊕⊝⊝ **low**
LDL Cholesterol (mmol/L)	WMD: 0.13 lower in the interventional group.	0.29 lower to 0.02 higher	157 (4 studies)	⊕⊕⊝⊝ **low**
Triglycerides (mmol/L)	WMD: 0.11 lower in the interventional group.	0.33 lower to 0.11 higher	157 (4 studies)	⊕⊕⊝⊝ **low**
Testosterone (nmol/L)	WMD: 0.04 lower in the interventional group.	0.41 lower to 0.32 higher	181 (4 studies)	⊕⊕⊝⊝ **low**
SHBG (nmol/L)	WMD: 4.65 higher in the interventional group.	5.36 lower to 14.66 higher	131 (3 studies)	⊕⊕⊝⊝ **low**
FAI	WMD: 1.53 lower in the interventional group.	3.11 lower to 0.05 higher	131 (3 studies)	⊕⊕⊝⊝ **low**

GRADE, Grading of Recommendations, Assessment, Development and Evaluation; WMD, Weighted mean differences; HIIT, High-intensity interval training; MICT, Moderate-intensity continuous training; CI, Confidence interval; BMI, Body mass index; WHR, Waist-to-hip ratio; SBP, Systolic blood pressure; DBP, Diastolic blood pressure; HOMA-IR, Homeostatic model assessment for insulin resistance; HDL, High-density lipoprotein; LDL, Low-density lipoprotein; SHBG, Sex hormone-binding globulin; FAI, Free androgen index.

GRADE Working Group grades of evidence

**High quality:** Further research is very unlikely to change our confidence in the estimate of effect.

**Moderate quality:** Further research is likely to have an important impact on our confidence in the estimate of effect and may change the estimate.

**Low quality:** Further research is very likely to have an important impact on our confidence in the estimate of effect and is likely to change the estimate.

**Very low quality:** We are very uncertain about the estimate.

## 4 Discussion

This meta-analysis synthesized current RCTs on the effects of HIIT versus MICT on women with PCOS. Our findings indicate that neither HIIT nor MICT demonstrated a statistically significant superiority over the other across anthropometric, cardiorespiratory, metabolic, or hormonal outcomes. Notably, the certainty of evidence for these outcomes ranged from very low to low, largely due to small sample sizes and inherent heterogeneity among studies.

Several recent systematic reviews have investigated exercise interventions in women with PCOS, each with distinct scopes and limitations. A 2021 review by Richards et al. (16) concluded Moderate-Intensity Steady State (MISS) exercise was superior for cardiorespiratory fitness and BMI in PCOS, but its scope was not limited to direct HIIT vs. MICT comparisons. Another 2021 review by Santos et al. (17) found HIIT alone significantly decreased HOMA-IR and BMI, but its primary objective was not head-to-head comparison of HIIT and MICT. A 2022 review by Breyley-Smith et al. (14) primarily compared exercise against non-exercising controls, concluding exercise improved cardiorespiratory fitness and waist circumference, with MICT showing greater or more significant improvements. Most recently, the 2023 review by Colombo et al. (15) found no statistically significant differences between HIIT and MICT for various parameters, with low or very low certainty of evidence. Notably, Colombo et al.’s meta-analysis included five RCTs. Three (28, 30, 32) directly compared HIIT and MICT (also in our review), while the other two compared HIIT to Resistance Training (RT) and MICT to MICT+RT.

To our best knowledge, our meta-analysis is the first to exclusively include RCTs directly comparing HIIT and MICT in women with PCOS. This rigorous focus on head-to-head comparisons, distinct from prior reviews that included broader exercise interventions or non-direct comparisons, ensures a more precise evaluation of their comparative effectiveness. Although the certainty of evidence for many outcomes remains very low to low due to limited studies and small sample sizes, our findings represent the highest quality evidence currently available in this specific comparative domain. Despite conclusions similar to Colombo et al. (15) regarding the lack of clear superiority, our expanded and updated dataset offers a more comprehensive and current perspective.

The observed absence of statistically significant superiority between HIIT and MICT, despite their distinct exercise protocols, can be largely attributed to a convergence in their underlying physiological mechanisms that target the core pathophysiological features of PCOS. Both modalities consistently improve insulin sensitivity, a cornerstone in PCOS management, by reducing markers such as fasting insulin and HOMA-IR (12, 13). This is crucial as it impacts the bidirectional link between hyperinsulinemia and hyperandrogenism, central to PCOS pathology (9–11). Beyond metabolic and hormonal improvements, both training types exert significant anti-inflammatory effects, mitigating chronic low-grade inflammation by modulating cytokine levels, which is vital for protecting against long-term cardiometabolic risks (14). In terms of body composition, both lead to favorable changes, including reductions in BMI, waist circumference, and overall body fat, while promoting increases in lean muscle mass (15–17). Last, both enhance cardiorespiratory fitness, indicated by improvements in maximal VO2max (15, 16). This broad spectrum of overlapping physiological benefits suggests that the body’s adaptive response to chronic exercise, irrespective of intensity, ultimately might converge on similar improvements in key PCOS indicators.

The “very low to low certainty of evidence” in our meta-analysis critically explains the lack of statistical superiority, primarily stemming from significant methodological limitations in existing RCTs. A key challenge is the inherent impossibility of participant blinding in exercise interventions, which invariably lowers the quality assessment of included studies. Another significant contributing factor is the consistently small sample sizes (typically 24–110 participants), reducing statistical power and hindering the detection of subtle yet clinically meaningful differences between HIIT and MICT. Heterogeneity across studies also manifests in variable designs, exercise protocols (intensity, duration, frequency, modality), participant characteristics (BMI, insulin resistance, PCOS phenotypes), and inconsistent outcome measurements. Finally, the relatively short duration of most studies (12–24 weeks) may be insufficient to reveal long-term or more significant differential effects in a chronic condition like PCOS.

Beyond statistical and methodological considerations, the practical implications of these findings warrant discussion. While our meta-analysis indicates no statistical superiority in efficacy, HIIT often achieves comparable physiological benefits in significantly less total exercise time compared to MICT. One study reported HIIT requiring 27.5% less total exercise time and approximately 25% less energy expenditure than MICT to achieve similar adaptations (33). This “time efficiency” is a crucial practical advantage that can significantly enhance patient adherence and long-term sustainability in real-world settings (34). However, a recent systematic review found that HIIT showed no advantage over MICT in unsupervised settings, as participants often exercised at lower-than-prescribed intensities (35). Despite our included studies being supervised and excluding those shorter than 12 weeks, these studies did not comprehensively report adherence, highlighting an important area for future research.

This meta-analysis has several limitations that warrant consideration. First, despite conducting a comprehensive search and including all eligible RCTs, the total number of studies and participants available for direct comparison between HIIT and MICT remains relatively small. Second, the heterogeneity in exercise protocols (such as exact intensity, duration of intervals, rest periods, overall weekly volume, and supervision levels) among the included studies, even within the broad categories of HIIT and MICT, makes it challenging to draw highly specific conclusions about optimal exercise prescription. This stems in part from methodological limitations of the review process, including our broad search strategy, which, while ensuring comprehensive capture of relevant studies, led to inclusion of trials with variations in practical parameters, with some minor adaptations as reported. Although inclusion criteria were consistently applied to verify direct HIIT versus MICT comparisons, these parameter variations may contribute to heterogeneity and compromise synthesis validity. Future meta-analyses could mitigate this by incorporating stricter subgroup analyses or protocol standardization. Third, while we aimed to isolate the effects of exercise, some studies included concurrent interventions like dietary advice, which, even if consistent across groups, could influence outcomes. Fourth, the majority of included studies involved women who were overweight or obese, and often had baseline cardiometabolic parameters within normal ranges. This limits the generalizability of our findings to lean women with PCOS or those with more severe metabolic abnormalities, where exercise might show more pronounced effects. Last, our review primarily focused on cardiometabolic and hormonal outcomes and did not extensively cover psychological aspects or long-term adherence beyond the intervention period, which are also crucial for comprehensive PCOS management.

## 5 Conclusion

Based on the current low to very low certainty evidence from RCTs, there is no statistically significant superiority of HIIT over MICT for improving anthropometric, cardiorespiratory, metabolic, or hormonal outcomes in women with PCOS. Given these findings, individuals with PCOS may select either HIIT or MICT based on personal preference and feasibility. Future large-scale, high-quality RCTs with standardized protocols and detailed reporting of participant phenotypes are needed.
