# Supplementary material for: High-intensity interval training versus moderate-intensity continuous training for polycystic ovary syndrome: a meta-analysis of randomized controlled trials
Source: Front Endocrinol (Lausanne). 2025 Oct 16;16:1672257. doi: 10.3389/fendo.2025.1672257 (PMC12571624; doi:10.3389/fendo.2025.1672257)
Supplement: Supplementary file 2 [file Table1.docx]

**Supplementary Table 1** Search terms for each database

| Database | Search terms |
| --- | --- |
| PubMed | ("High-Intensity Interval Training"[MeSH Terms] OR "high-intensity interval training" OR "HIIT" OR "high-intensity intermittent exercise" OR "interval training") AND ("Exercise"[MeSH Terms] OR "moderate-intensity continuous training" OR "MCIT" OR "moderate-intensity exercise" OR "continuous aerobic training") AND ("Polycystic Ovary Syndrome"[MeSH Terms] OR "polycystic ovary syndrome" OR "PCOS" OR "polycystic ovarian syndrome" OR "hyperandrogenic anovulation") |
| EMBASE | ('high intensity interval training'/exp OR 'high-intensity interval training' OR 'HIIT' OR 'high-intensity intermittent exercise' OR 'interval training') AND ('moderate intensity continuous training'/exp OR 'moderate-intensity continuous training' OR 'MCIT' OR 'moderate-intensity exercise' OR 'continuous aerobic training') AND ('polycystic ovary syndrome'/exp OR 'polycystic ovary syndrome' OR 'PCOS' OR 'polycystic ovarian syndrome' OR 'hyperandrogenic anovulation') |
| Cochrane Library | ("high-intensity interval training" OR "HIIT" OR "high-intensity intermittent exercise" OR "interval training") AND ("moderate-intensity continuous training" OR "MCIT" OR "moderate-intensity exercise" OR "continuous aerobic training") AND ("polycystic ovary syndrome" OR "PCOS" OR "polycystic ovarian syndrome" OR "hyperandrogenic anovulation") in All Text |
| Web of Science | ("high-intensity interval training" OR "HIIT" OR "high-intensity intermittent exercise" OR "interval training") AND ("moderate-intensity continuous training" OR "MCIT" OR "moderate-intensity exercise" OR "continuous aerobic training") AND ("polycystic ovary syndrome" OR "PCOS" OR "polycystic ovarian syndrome" OR "hyperandrogenic anovulation") |
